# {3-[(2-Chloro-1,3-thia­zol-4-yl)meth­yl]-1,3-thia­zolidin-2-yl­idene­amino}­formonitrile

**DOI:** 10.1107/S1600536810035579

**Published:** 2010-09-11

**Authors:** Ying-qi Li

**Affiliations:** a Vocational–Technical Institute, Xiangtan University, Xiangtan 411100, People’s Republic of China

## Abstract

In the title compound, C_8_H_7_ClN_4_S_2_, the dihedral angle between the thia­zolidine ring (r.m.s. deviation = 0.028 Å) and the thia­zole ring (r.m.s. deviation = 0.004 Å) is 74.74 (6)°. The formonitrile group is almost coplanar with the attached ring [C—N—C—N torsion angle =  167 (2)°.

## Related literature

For the biological activity of compounds containing a thia­zole ring, see: Ehrenfreund *et al.* (2003 ▶); Kim *et al.* (2002 ▶); Maienfisch & Gsell (1998 ▶); Shiga *et al.* (2003 ▶); Smith & Hunter (2001 ▶); Tanaka *et al.* (2005 ▶). For the bioactivity of 1,3-thia­zolidine derivatives, see: Albrecht *et al.* (2005 ▶); Liu & Li (2000 ▶); Ueda *et al.* (2004 ▶); Yeh & Chen (2002 ▶).
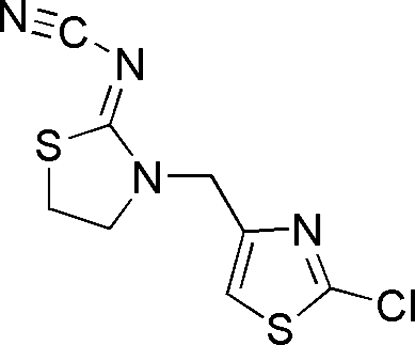


## Experimental

### 

#### Crystal data

C_8_H_7_ClN_4_S_2_
*M* *_r_* = 258.75
Monoclinic, 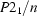

*a* = 6.1731 (9) Å
*b* = 16.807 (2) Å
*c* = 10.9057 (14) Å
β = 105.846 (2)°
*V* = 1088.5 (3) Å^3^
*Z* = 4
Mo *K*α radiation
μ = 0.70 mm^−1^
*T* = 294 K
0.22 × 0.20 × 0.18 mm

#### Data collection

Bruker SMART CCD area-detector diffractometer
Absorption correction: multi-scan (*SADABS*; Sheldrick, 1996 ▶) *T*_min_ = 0.860, *T*_max_ = 0.884
6141 measured reflections
2220 independent reflections
1862 reflections with *I* > 2σ(*I*)
*R*_int_ = 0.021

#### Refinement

*R*[*F* ^2^ > 2σ(*F* ^2^)] = 0.036
*wR*(*F* ^2^) = 0.103
*S* = 1.05
2220 reflections
136 parameters
H-atom parameters constrained
Δρ_max_ = 0.30 e Å^−3^
Δρ_min_ = −0.34 e Å^−3^

### 

Data collection: *SMART* (Bruker, 2000 ▶); cell refinement: *SAINT* (Bruker, 2000 ▶); data reduction: *SAINT*; program(s) used to solve structure: *SHELXS97* (Sheldrick, 2008 ▶); program(s) used to refine structure: *SHELXL97* (Sheldrick, 2008 ▶); molecular graphics: *SHELXTL* (Sheldrick, 2008 ▶); software used to prepare material for publication: *SHELXTL*.

## Supplementary Material

Crystal structure: contains datablocks global, I. DOI: 10.1107/S1600536810035579/ci5173sup1.cif

Structure factors: contains datablocks I. DOI: 10.1107/S1600536810035579/ci5173Isup2.hkl

Additional supplementary materials:  crystallographic information; 3D view; checkCIF report

## Figures and Tables

**Fig. 1.**
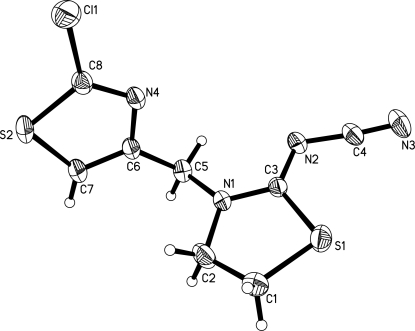
The molecular structure of the title compound, showing the atom-numbering scheme. Displacement ellipsoids are drawn at the 30% probability level.

## supplementary crystallographic information

### Comment

Recently, compounds containing thiazole ring have been reported to possess various biological activities such as fungicidal, insecticidal, and anticancer activities (Maienfisch & Gsell, 1998; Smith *et al.*, 2001; Kim *et al.*, 2002; Ehrenfreund *et al.*, 2003; Shiga *et al.*, 2003; Tanaka *et al.*, 2005). In addition, 1,3-thiazolidine ring is an important heterocycle scaffold among thiazole compounds. In the past few years lots of 1,3-thiazolidine derivatives have attracted intense attention in medicinal research due to their broad spectrum bioactivities (Liu *et al.*, 2000; Yeh *et al.*, 2002; Ueda *et al.*, 2004;Albrecht *et al.*, 2005). In order to discover more biologically active thiazole compounds, we synthesized thiazole compounds containing 1,3-thiazolidine ring and we report here the crystal structure of the title compound.

The molecule of the title compound (Fig.1) contains two planar rings, the substituted 1,3-thiazolidine ring (S1/C1/C2/N1/C3, r.m.s. deviation 0.028 Å) and the thiazole ring (S2/C8/N4/C6/C7, r.m.s. deviation 0.004 Å). The dihedral angle between the planes of 1,3-thiazolidine ring and thiazole ring is 74.74 (6)°.

### Experimental

To a stirred solution of 2-cyanoimino-1,3-thiazolidine (1.27 g, 0.01 mol), potassium carbonate (1.66 g,0.012 mol) in 20 ml of acetonitrile was added dropwise a solution of 2-chloro-4-(chloromethyl)thiazole (1.68 g, 0.01 mol) in 15 ml of acetonitrile. The reaction mixture was heated to 333 K for 12 h and then filtered. The solvent was removed to give a solid product, which was recrystallized from ethyl acetate to afford colourless crystals.

### Refinement

All H atoms were placed in calculated positions, with C–H = 0.93 and 0.97 Å, and included in the final cycles of refinement using a riding model, with *U*_iso_(H) = 1.2*U*_eq_(C).

### Crystal data

**Table:**

C_8_H_7_ClN_4_S_2_	*F*(000) = 528
*M**_r_* = 258.75	*D*_x_ = 1.579 Mg m^−^^3^
Monoclinic, *P*2_1_/*n*	Mo *K*α radiation, λ = 0.71073 Å
Hall symbol: -P 2yn	Cell parameters from 3431 reflections
*a* = 6.1731 (9) Å	θ = 2.4–26.2°
*b* = 16.807 (2) Å	µ = 0.70 mm^−^^1^
*c* = 10.9057 (14) Å	*T* = 294 K
β = 105.846 (2)°	Monoclinic, colourless
*V* = 1088.5 (3) Å^3^	0.22 × 0.20 × 0.18 mm
*Z* = 4	

### Data collection

**Table:**

Bruker SMART CCD area-detector diffractometer	2220 independent reflections
Radiation source: fine-focus sealed tube	1862 reflections with *I* > 2σ(*I*)
graphite	*R*_int_ = 0.021
φ and ω scans	θ_max_ = 26.4°, θ_min_ = 2.3°
Absorption correction: multi-scan (*SADABS*; Sheldrick, 1996)	*h* = −7→7
*T*_min_ = 0.860, *T*_max_ = 0.884	*k* = −15→21
6141 measured reflections	*l* = −11→13

### Refinement

**Table:**

Refinement on *F*^2^	Primary atom site location: structure-invariant direct methods
Least-squares matrix: full	Secondary atom site location: difference Fourier map
*R*[*F*^2^ > 2σ(*F*^2^)] = 0.036	Hydrogen site location: inferred from neighbouring sites
*wR*(*F*^2^) = 0.103	H-atom parameters constrained
*S* = 1.05	*w* = 1/[σ^2^(*F*_o_^2^) + (0.0573*P*)^2^ + 0.3256*P*] where *P* = (*F*_o_^2^ + 2*F*_c_^2^)/3
2220 reflections	(Δ/σ)_max_ = 0.001
136 parameters	Δρ_max_ = 0.30 e Å^−^^3^
0 restraints	Δρ_min_ = −0.34 e Å^−^^3^

### Special details

**Table:**

Geometry. All e.s.d.'s (except the e.s.d. in the dihedral angle between two l.s. planes) are estimated using the full covariance matrix. The cell e.s.d.'s are taken into account individually in the estimation of e.s.d.'s in distances, angles and torsion angles; correlations between e.s.d.'s in cell parameters are only used when they are defined by crystal symmetry. An approximate (isotropic) treatment of cell e.s.d.'s is used for estimating e.s.d.'s involving l.s. planes.
Refinement. Refinement of *F*^2^ against ALL reflections. The weighted *R*-factor *wR* and goodness of fit *S* are based on *F*^2^, conventional *R*-factors *R* are based on *F*, with *F* set to zero for negative *F*^2^. The threshold expression of *F*^2^ > σ(*F*^2^) is used only for calculating *R*-factors(gt) *etc*. and is not relevant to the choice of reflections for refinement. *R*-factors based on *F*^2^ are statistically about twice as large as those based on *F*, and *R*- factors based on ALL data will be even larger.

### Fractional atomic coordinates and isotropic or equivalent isotropic displacement parameters (Å^2^)

**Table:**

	*x*	*y*	*z*	*U*_iso_*/*U*_eq_	
Cl1	−0.27073 (11)	0.02267 (4)	0.65605 (6)	0.0705 (2)	
S1	0.70384 (10)	0.09234 (4)	1.21031 (5)	0.0588 (2)	
S2	0.07788 (10)	0.10824 (3)	0.56567 (5)	0.05097 (18)	
N1	0.4957 (3)	0.15672 (9)	0.99768 (14)	0.0389 (4)	
N2	0.3327 (3)	0.18765 (11)	1.15862 (15)	0.0466 (4)	
N3	0.3408 (4)	0.18457 (14)	1.38596 (19)	0.0669 (6)	
N4	0.0452 (3)	0.11665 (10)	0.79535 (15)	0.0410 (4)	
C1	0.8149 (4)	0.07222 (18)	1.0769 (2)	0.0660 (7)	
H1A	0.9724	0.0873	1.0975	0.079*	
H1B	0.8034	0.0159	1.0571	0.079*	
C2	0.6843 (4)	0.11841 (17)	0.9667 (2)	0.0631 (7)	
H2A	0.7801	0.1583	0.9440	0.076*	
H2B	0.6296	0.0834	0.8942	0.076*	
C3	0.4872 (3)	0.15132 (11)	1.11773 (17)	0.0370 (4)	
C4	0.3448 (4)	0.18300 (13)	1.2819 (2)	0.0478 (5)	
C5	0.3492 (3)	0.20877 (12)	0.90364 (17)	0.0426 (4)	
H5A	0.4394	0.2499	0.8790	0.051*	
H5B	0.2428	0.2346	0.9417	0.051*	
C6	0.2233 (3)	0.16430 (11)	0.78796 (17)	0.0378 (4)	
C7	0.2661 (4)	0.16624 (12)	0.67255 (17)	0.0451 (5)	
H7	0.3818	0.1951	0.6541	0.054*	
C8	−0.0433 (3)	0.08538 (11)	0.68491 (19)	0.0428 (4)	

### Atomic displacement parameters (Å^2^)

**Table:**

	*U*^11^	*U*^22^	*U*^33^	*U*^12^	*U*^13^	*U*^23^
Cl1	0.0659 (4)	0.0730 (4)	0.0743 (4)	−0.0187 (3)	0.0221 (3)	−0.0253 (3)
S1	0.0642 (4)	0.0707 (4)	0.0407 (3)	0.0175 (3)	0.0133 (3)	0.0086 (3)
S2	0.0674 (4)	0.0577 (3)	0.0290 (3)	0.0066 (3)	0.0152 (2)	−0.0030 (2)
N1	0.0391 (8)	0.0492 (9)	0.0307 (8)	0.0022 (7)	0.0131 (6)	−0.0022 (7)
N2	0.0483 (9)	0.0586 (10)	0.0382 (9)	0.0007 (8)	0.0208 (7)	−0.0049 (7)
N3	0.0869 (15)	0.0758 (14)	0.0499 (11)	−0.0106 (11)	0.0390 (11)	−0.0082 (10)
N4	0.0470 (9)	0.0462 (9)	0.0335 (8)	0.0025 (7)	0.0174 (7)	−0.0024 (7)
C1	0.0546 (13)	0.0878 (18)	0.0591 (14)	0.0197 (13)	0.0218 (11)	0.0030 (13)
C2	0.0664 (15)	0.0811 (17)	0.0517 (13)	0.0266 (13)	0.0328 (12)	0.0063 (12)
C3	0.0369 (9)	0.0420 (10)	0.0328 (9)	−0.0068 (8)	0.0108 (7)	−0.0030 (7)
C4	0.0526 (12)	0.0519 (12)	0.0465 (12)	−0.0061 (9)	0.0264 (9)	−0.0056 (9)
C5	0.0517 (11)	0.0417 (10)	0.0356 (9)	0.0012 (8)	0.0140 (8)	−0.0005 (8)
C6	0.0448 (10)	0.0398 (10)	0.0310 (9)	0.0074 (8)	0.0141 (7)	0.0027 (7)
C7	0.0549 (12)	0.0498 (11)	0.0345 (10)	0.0026 (9)	0.0191 (9)	0.0019 (8)
C8	0.0470 (10)	0.0435 (10)	0.0397 (10)	0.0051 (8)	0.0149 (8)	−0.0041 (8)

### Geometric parameters (Å, °)

**Table:**

Cl1—C8	1.715 (2)	N4—C6	1.380 (2)
S1—C3	1.7471 (19)	C1—C2	1.472 (3)
S1—C1	1.801 (2)	C1—H1A	0.97
S2—C7	1.709 (2)	C1—H1B	0.97
S2—C8	1.711 (2)	C2—H2A	0.97
N1—C3	1.328 (2)	C2—H2B	0.97
N1—C2	1.448 (3)	C5—C6	1.490 (3)
N1—C5	1.459 (2)	C5—H5A	0.97
N2—C3	1.309 (2)	C5—H5B	0.97
N2—C4	1.328 (3)	C6—C7	1.355 (3)
N3—C4	1.142 (3)	C7—H7	0.93
N4—C8	1.291 (2)		
			
C3—S1—C1	92.29 (10)	N2—C3—N1	122.18 (17)
C7—S2—C8	88.08 (10)	N2—C3—S1	125.57 (14)
C3—N1—C2	116.74 (17)	N1—C3—S1	112.25 (14)
C3—N1—C5	123.40 (16)	N3—C4—N2	173.6 (3)
C2—N1—C5	119.09 (15)	N1—C5—C6	111.98 (15)
C3—N2—C4	118.19 (18)	N1—C5—H5A	109.2
C8—N4—C6	108.81 (16)	C6—C5—H5A	109.2
C2—C1—S1	108.40 (16)	N1—C5—H5B	109.2
C2—C1—H1A	110.0	C6—C5—H5B	109.2
S1—C1—H1A	110.0	H5A—C5—H5B	107.9
C2—C1—H1B	110.0	C7—C6—N4	115.37 (17)
S1—C1—H1B	110.0	C7—C6—C5	125.85 (19)
H1A—C1—H1B	108.4	N4—C6—C5	118.78 (16)
N1—C2—C1	109.89 (18)	C6—C7—S2	110.57 (16)
N1—C2—H2A	109.7	C6—C7—H7	124.7
C1—C2—H2A	109.7	S2—C7—H7	124.7
N1—C2—H2B	109.7	N4—C8—S2	117.15 (16)
C1—C2—H2B	109.7	N4—C8—Cl1	122.55 (16)
H2A—C2—H2B	108.2	S2—C8—Cl1	120.29 (12)
			
C3—S1—C1—C2	4.2 (2)	C3—N1—C5—C6	−124.90 (19)
C3—N1—C2—C1	6.9 (3)	C2—N1—C5—C6	65.5 (2)
C5—N1—C2—C1	177.2 (2)	C8—N4—C6—C7	−1.2 (2)
S1—C1—C2—N1	−6.7 (3)	C8—N4—C6—C5	178.42 (17)
C4—N2—C3—N1	−175.95 (18)	N1—C5—C6—C7	−106.0 (2)
C4—N2—C3—S1	3.3 (3)	N1—C5—C6—N4	74.4 (2)
C2—N1—C3—N2	175.7 (2)	N4—C6—C7—S2	0.9 (2)
C5—N1—C3—N2	5.9 (3)	C5—C6—C7—S2	−178.68 (15)
C2—N1—C3—S1	−3.7 (2)	C8—S2—C7—C6	−0.28 (16)
C5—N1—C3—S1	−173.46 (14)	C6—N4—C8—S2	1.0 (2)
C1—S1—C3—N2	−179.83 (19)	C6—N4—C8—Cl1	−179.65 (14)
C1—S1—C3—N1	−0.49 (17)	C7—S2—C8—N4	−0.42 (17)
C3—N2—C4—N3	167 (2)	C7—S2—C8—Cl1	−179.83 (14)
